# Predicting Mortality in Subarachnoid Hemorrhage Patients Using Big Data and Machine Learning: A Nationwide Study in Türkiye

**DOI:** 10.3390/jcm14041144

**Published:** 2025-02-10

**Authors:** Taghi Khaniyev, Efecan Cekic, Neslihan Nisa Gecici, Sinem Can, Naim Ata, Mustafa Mahir Ulgu, Suayip Birinci, Ahmet Ilkay Isikay, Abdurrahman Bakir, Anil Arat, Sahin Hanalioglu

**Affiliations:** 1 Department of Industrial Engineering, Faculty of Engineering, Bilkent University, 06800 Ankara, Türkiye; taghi.khaniyev@bilkent.edu.tr; 2 National Magnetic Resonance Research Center (UMRAM), Bilkent University, 06800 Ankara, Türkiye; 3 Sloan School of Management, Massachusetts Institute of Technology, Cambridge, MA 02139, USA; 4 Department of Neurosurgery, Faculty of Medicine, Hacettepe University, 06100 Ankara, Türkiye; drefecancekic@gmail.com (E.C.); n.ngecici@gmail.com (N.N.G.); ilkayisikay@gmail.com (A.I.I.); 5 Department of Neurological Surgery, University of Pittsburgh School of Medicine, Pittsburgh, PA 15213, USA; 6 General Directorate of Health Information System, Republic of Türkiye Ministry of Health, 06800 Ankara, Türkiye; sinem.can@saglik.gov.tr (S.C.); naim.ata@saglik.gov.tr (N.A.); mahir.ulgu@saglik.gov.tr (M.M.U.); 7 Republic of Türkiye Ministry of Health, 06800 Ankara, Türkiye; suayipbirinci@gmail.com; 8 Department of Neurosurgery, Dr. Abdurrahman Yurtaslan Oncology Research and Education Hospital, 06800 Ankara, Türkiye; abdbak@hotmail.com; 9 Department of Radiology, Faculty of Medicine, Hacettepe University, 06230 Ankara, Türkiye; anilarat@hotmail.com; 10 Department of Neurosurgery, School of Medicine, Yale University, New Haven, CT 06520, USA; 11 Department of Neurosurgery, Brigham and Women’s Hospital, Harvard Medical School, Boston, MA 02115, USA

**Keywords:** subarachnoid hemorrhage, machine learning, mortality prediction, logistic regression, artificial neural network, artificial intelligence

## Abstract

**Background/Objective:** Subarachnoid hemorrhage (SAH) is associated with high morbidity and mortality rates, necessitating prognostic algorithms to guide decisions. Our study evaluates the use of machine learning (ML) models for predicting 1-month and 1-year mortality among SAH patients using national electronic health records (EHR) system. **Methods:** Retrospective cohort of 29,274 SAH patients, identified through national EHR system from January 2017 to December 2022, was analyzed, with mortality data obtained from central civil registration system in Türkiye. Variables included (*n* = 102) pre- (*n* = 65) and post-admission (*n* = 37) data, such as patient demographics, clinical presentation, comorbidities, laboratory results, and complications. We employed logistic regression (LR), decision trees (DTs), random forests (RFs), and artificial neural networks (ANN). Model performance was evaluated using area under the curve (AUC), average precision, and accuracy. Feature significance analysis was conducted using LR. **Results:** The average age was 56.23 ± 16.45 years (47.8% female). The overall mortality rate was 22.8% at 1 month and 33.3% at 1 year. One-month mortality increased from 20.9% to 24.57% (*p* < 0.001), and 1-year mortality rose from 30.85% to 35.55% (*p* < 0.001) in the post-COVID period compared to the pre-COVID period. For 1-month mortality prediction, the ANN, LR, RF, and DT models achieved AUCs of 0.946, 0.942, 0.931, and 0.916, with accuracies of 0.905, 0.901, 0.893, and 0.885, respectively. For 1-year mortality, the AUCs were 0.941, 0.927, 0.926, and 0.907, with accuracies of 0.884, 0.875, 0.861, and 0.851, respectively. Key predictors of mortality included age, cardiopulmonary arrest, abnormal laboratory results (such as abnormal glucose and lactate levels) at presentation, and pre-existing comorbidities. Incorporating post-admission features (*n* = 37) alongside pre-admission features (*n* = 65) improved model performance for both 1-month and 1-year mortality predictions, with average AUC improvements of 0.093 ± 0.011 and 0.089 ± 0.012, respectively. **Conclusions:** Our study demonstrates the effectiveness of ML models in predicting mortality in SAH patients using big data. LR models’ robustness, interpretability, and feature significance analysis validate its importance. Including post-admission data significantly improved all models’ performances. Our results demonstrate the utility of big data analytics in population-level health outcomes studies.

## 1. Introduction

Subarachnoid hemorrhage (SAH) is a severe, acute neurological disorder characterized by bleeding into the subarachnoid space, often leading to significant morbidity and mortality, especially among patients with poor neurological grade at admission [1,2,3]. The SAH continues to be an essential public health problem, as patients usually arrive at emergency departments with severe neurological deterioration [4,5]. Timely prognosis prediction in patients with SAH is necessary for optimizing clinical care and allocating resources effectively, potentially enhancing patient outcomes and reducing hospital costs [6,7].

Recent machine learning (ML) advances have shown promise in predicting outcomes for various neurological conditions in surgical patients [8,9], including SAH. Studies have demonstrated the utility of decision tree (DT) models for predicting long-term outcomes after poor-grade aneurysmal SAH [10]. Moreover, complication and treatment-aware ML models have also shown improved prediction accuracy for functional consequences [11]. Metabolic evaluation of cerebrospinal fluid (CSF) and plasma for predicting SAH prognosis and delayed cerebral ischemia (DCI) has also provided insights into poor outcomes [12,13,14]. Artificial neural networks (ANNs) have been applied to predict clinical outcomes and DCI [15,16].

Despite extensive research, there remains a need for large-scale studies specifically targeting mortality predictions and poor prognosis factors in SAH patients. Türkiye’s e-Nabız system offers a comprehensive resource, encompassing nationwide electronic health records (EHR) from all hospitals in the country with a population of over 85 million. This extensive platform provides a unique opportunity to study disease-specific healthcare outcomes at the population level. Our study leverages this robust database to enhance the validity and applicability of our findings across all the geographical regions in Türkiye, marking it the first comprehensive nationwide analysis.

We aim to utilize big data extracted via a nationwide EHR to develop predictive models for 1-month and 1-year mortality in SAH patients, identifying critical prognostic factors using advanced ML algorithms. The dataset’s comprehensiveness will allow us to integrate both pre- and post-admission clinical variables, enabling the identification of high-risk patients and parameters. This approach will facilitate the development of tailored interventions, ultimately improving patient outcomes, optimizing hospital resource allocation, and reducing healthcare costs. By applying ML techniques such as logistic regression (LR), decision trees (DTs), random forests (RFs), and ANNs, we seek to provide a nuanced understanding of SAH prognosis and enhance clinical decision-making.

## 2. Method

Our study was conducted with the approval of the Ministry of Health of the Republic of Türkiye, utilizing the e-Nabız system, which serves as Türkiye’s comprehensive national health information platform. Patient consent was not required, as the study was retrospective, and the data were anonymized. The e-Nabız system contains essential patient information, including diagnoses, imaging findings, examination, and treatment history. All methods were conducted in accordance with relevant guidelines and regulations. The dataset used in the analysis was acquired in compliance with the Personal Data Protection Law No. 6698, dated 24 March 2016, of the Republic of Türkiye. The data acquisition and analysis were also conducted according to the Ethics Committee for Public Servants guidelines under the Ministry’s Ethics Commission. The study was reviewed by commission officers, who confirmed that all data collection, analysis, and reporting processes adhered to scientific and ethical standards. The mortality times of patients were extracted from the Central Civil Registration System (MERNİS). The MERNİS system is considered a robust and reliable source for obtaining accurate mortality data in Türkiye.

### 2.1. Patient Selection

The study population comprised patients diagnosed with spontaneous SAH, confirmed using the ICD-10 code I60 (subarachnoid hemorrhage), who presented to emergency departments (EDs) in Türkiye between January 2017 and December 2022. To be eligible for inclusion, patients were required to have a confirmed SAH diagnosis and had to have undergone a computed tomography (CT) scan. Exclusion criteria included patients with traumatic SAH and incomplete medical records, specifically those lacking essential data points such as imaging results or follow-up information. Instances with unclear diagnostic codes or those lacking confirmatory imaging were also eliminated.

### 2.2. Feature Selection and Data Preprocessing

Feature selection was based on previous studies and clinical relevance, identifying a broad set of potential predictors. Comorbidities were identified based on ICD codes documented before the index emergency visit, while complications were identified through new ICD codes recorded after the initial emergency visit.

The pre-admission data include comprehensive clinical information collected from patients upon arrival at the ED. This dataset (*n* = 65 features) captures initial patient demographics, clinical presentations, and immediate laboratory results obtained during the ED visit. It encompasses critical early assessments such as the patient’s first-present condition, initial vital signs, and laboratory tests, including a complete blood count (CBC), basic metabolic panel, and blood gases. Additionally, pre-existing medical conditions documented in the patient’s history are included to provide a baseline for subsequent analysis.

The post-admission data encompass all clinical observations and outcomes recorded after patients were formally admitted to the hospital. This dataset (*n* = 37 features) includes detailed information on the medical interventions received, surgical or endovascular treatments, complications (e.g., infections, neurological events, cardiopulmonary conditions), and their management during hospitalization.

The study utilized these distinct data phases to develop predictive models. The pre-admission data provide a snapshot of the patient’s condition at initial medical contact, capturing early diagnostic indicators and potential risk factors. In contrast, the post-admission data offer a longitudinal view of patient outcomes and the impact of various interventions or management strategies, including detailed complications tracking and assessments of treatment efficacy. By incorporating both datasets, the analysis aims to comprehensively assess factors influencing patient prognosis, from initial presentation through hospitalization.

Data preprocessing involved applying a min–max scaler, where each feature was linearly transformed such that the minimum value was set to 0 and the maximum value to 1. This scaling and one-hot encoding of categorical variables were implemented to enhance the data’s compatibility with ML algorithms. Including pre- and post-admission parameters data in the predictive models was a deliberate decision to comprehend the broader spectrum of patient data, thereby improving the models’ predictive power.

### 2.3. Machine Learning Techniques

Two sets of predictive models were created to predict the likelihood of mortality at one month and one year after the index event of SAH. One set included pre-admission characteristics; the other incorporated pre- and post-admission parameters. The pre-admission model included age, gender, initial clinical presentation, and early laboratory findings. In contrast, the post-admission model included characteristics of the interventional process and post-admission neurological, laboratory, and clinical features.

Four ML techniques were utilized: LR, DT, RF, and ANN. Each model’s training and test employed a dataset split of 70:30, where 70% was allocated for training and 30% for testing. Cross-validation was conducted to optimize model hyperparameters and mitigate overfitting. The four models’ performance was assessed on test samples using AUC, average precision (AP), and accuracy, aligned with previous studies leveraging machine learning for predictive modeling in clinical outcome research [17,18,19,20,21].

(i) Logistic regression (LR) is a basic yet robust prediction algorithm that estimates the probability of a binary outcome based on a set of input features. It calculates a weighted sum of the input features using coefficients learned during training, then applies a logistic function to constrain the output to a range of 0 to 1. This value represents the likelihood of the positive class or event (in our case, “death within 1 month” and “death within 1 year”).

(ii) Decision trees (DT) work by recursively splitting the dataset into subsets based on specific feature values, forming a tree-like structure. Each split condition, represented by a branching node, determines the direction in which data points flow, either left or right. The terminal leaf nodes signify the final outcome, where the predicted class is derived from the majority class of the training data points at that leaf.

(iii) Random forest (RF) is an ensemble learning approach that builds multiple decision trees using random subsets of the training data and input features. The model aggregates the predictions from these individual trees, typically by averaging or voting, to generate the final output. This ensemble strategy enhances the model’s accuracy and resilience by mitigating the tendency to overfit.

(iv) Artificial neural networks (ANNs) are the simplest type of neural networks, where information flows in a single direction—from the input layer (representing features) through one or more hidden layers (where learning occurs) to the output layer (indicating the predicted outcome). Hidden layers are composed of interconnected nodes that perform linear transformations on their inputs, followed by nonlinear activation functions, enabling the network to identify and learn complex patterns in the training data.

### 2.4. Statistical Analysis

Descriptive statistics for parametric continuous variables were presented as means and standard deviations (±), while non-parametric continuous variables were summarized using medians, first and third quartiles [Q1–Q3]. Categorical variables were summarized as numbers (percentages, [*n* (%)]). Logistic regression was employed to determine the most influential predictors, and the statistical significance of each variable was evaluated using *p*-values. Variables with *p*-values below 0.05 were deemed statistically significant. Variables with greater significance were classified into different categories depending on their *p*-values: (i) *p* < 0.001, (ii) *p* < 0.01, and (iii) *p* < 0.05.

We used Python (version 3.8) for statistical studies with Scikit-learn (version 1.3.2) and R (version 4.2.1) for statistical significance analysis. We evaluated different models by analyzing their performance indicators, choosing the model with the highest AUC score as the best-performing one.

## 3. Results

### 3.1. Patient Characteristics

As detailed in Table 1, this study included 29,274 patients diagnosed with SAH between January 2017 and December 2022. Our cohort had a median age of 56.23 ± 16.45 years, with 52.2% male and 47.8% female patients. The median time from the initial emergency visit to the CT scan was 12 [4–114] minutes. Notably, 34.2% of the patients experienced cardiopulmonary arrests and/or necessitated endotracheal intubation within the first 24 h, and 9.7% required external ventricular drain (EVD) placement within the first 72 h. Initial laboratory values at emergency presentation included a median hemoglobin level of 13 [11.4–14.4] g/dL, a white blood cell count of 12.01 × 10^3^ [9.22–15.6] cells/μL, and 131 [105–167] mg/dL blood glucose level. The cohort also exhibited comorbidities, including hypertension (55.7%), chronic ischemic heart disease (26.8%), and type 2 diabetes mellitus (25.5%). The results indicate that 8220 patients (28.1%) were diagnosed with epilepsy. The average length of hospitalization was approximately 19.01 ± 15.94 days. The mortality rates within the first week, the first month, and the first year were 9.4%, 22.8%, and 33.3%, respectively.

Out of the total cohort, 37.8% of patients underwent primary intervention for aneurysms, with 22.7% undergoing clipping and 15.1% undergoing coiling. The median time from the initial emergency visit to intervention was 1.95 days [0.75–4.75 days], with clipping occurring at a median of 1.95 days [0.77–4.4 days] and coiling at 2.07 days [0.8–5.8 days].

Regarding the institutions where interventions for aneurysms were performed, the majority occurred in government-owned hospitals (49.3%), followed by university hospitals (30.7%). Finally, 19.7% were carried out in private institutions. This distribution highlights the significant role of public healthcare institutions in managing aneurysmal interventions within the studied cohort.

### 3.2. First-Month Mortality Prediction

**Figure 1:**
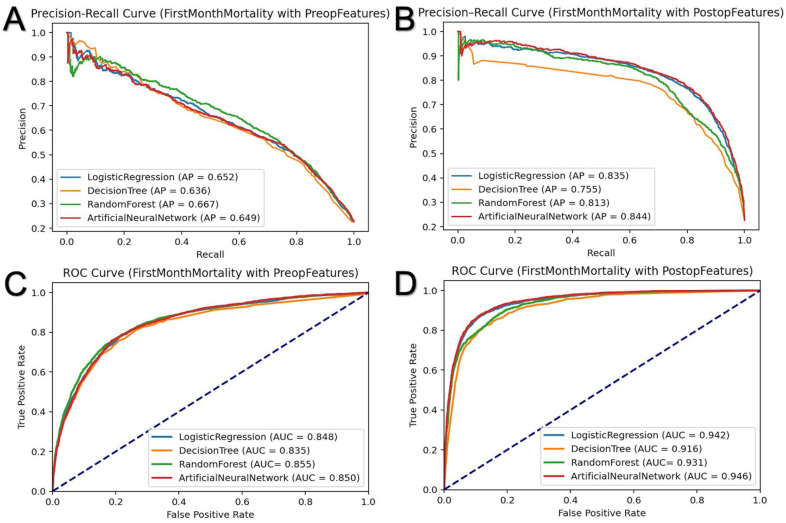
Precision–recall and Receiver Operating Characteristic (ROC) curves for predicting mortality in subarachnoid hemorrhage patients. Using various feature sets, the curves illustrate the predictive performance of different machine learning models for first-month mortality outcomes. (**A**) shows the precision–recall curve for first-month mortality prediction using preoperative features alone. (**B**) presents the precision–recall curve for first-month mortality prediction incorporating both postoperative and preoperative features. (**C**) displays the ROC curve for first-month mortality prediction using only preoperative features. (**D**) highlights the ROC curve for first-month mortality prediction with the addition of postoperative features.

The first-month mortality rate in our cohort was 22.8%. As depicted in Table 2 and Figure 1, in the test sets, the pre-admission LR model achieved an AUC of 0.849, an average precision of 0.651, and an accuracy of 0.832. The DT model had an AUC of 0.835, an average precision of 0.636, and an accuracy of 0.826. The RF model demonstrated an AUC of 0.855, an average precision of 0.667, and an accuracy of 0.835. The ANN model achieved an AUC of 0.850, an average precision of 0.649, and an accuracy of 0.829.

When post-admission parameters were included, the predictive performance of the models significantly improved in the test sets. The LR model’s AUC increased to 0.942, with an average precision of 0.835 and an accuracy of 0.901. The DT model achieved an AUC of 0.916, an average precision of 0.755, and an accuracy of 0.885. The RF model’s AUC rose to 0.931, with an average precision of 0.813 and an accuracy of 0.893. The ANN model demonstrated the best performance, with an AUC of 0.946, an average precision of 0.844, and an accuracy of 0.905.

In addition to pre-admission features (*n* = 65), post-admission features (*n* = 37) significantly improved the model’s performance in terms of 1-month mortality prediction. The AUC for the ANN model increased from 0.850 to 0.946, RF from 0.855 to 0.931, DT from 0.835 to 0.916, and LR from 0.849 to 0.942. On average, the AUC improvement across all models was 0.093 ± 0.011, reflecting a consistent enhancement across all methods.

### 3.3. First-Year Mortality Prediction

**Figure 2:**
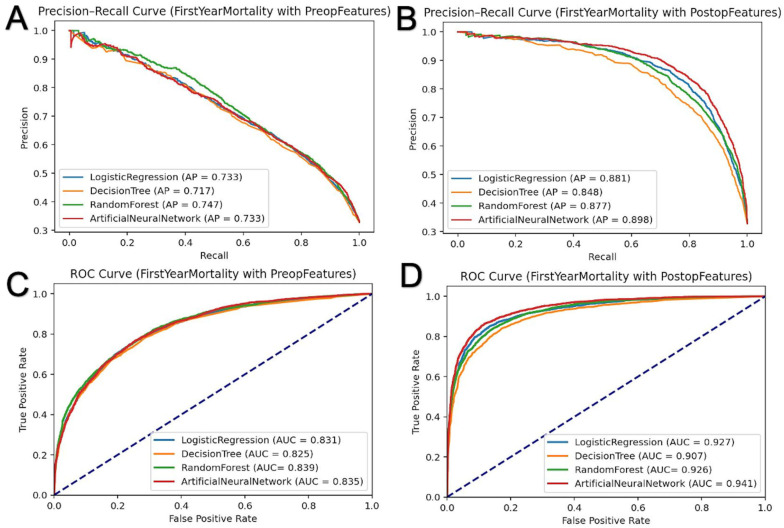
Precision–recall and Receiver Operating Characteristic (ROC) curves for predicting first-year mortality in subarachnoid hemorrhage patients. Using various feature sets, the curves illustrate the predictive performance of different machine learning models for first-year mortality outcomes. (**A**) shows the precision–recall curve for first-year mortality prediction using preoperative features alone. (**B**) presents the precision–recall curve for first-year mortality prediction incorporating both postoperative and preoperative features. (**C**) displays the ROC curve for first-year mortality prediction using only preoperative features, while (**D**) highlights the ROC curve for first-year mortality prediction by adding postoperative features.

The first-year mortality rate in our cohort was 33.3%. As shown in Table 3 and Figure 2, similar models were developed to predict first-year mortality. In the test sets, the pre-admission LR model achieved an AUC of 0.831, an average precision of 0.733, and an accuracy of 0.782. The DT model had an AUC of 0.825, an average precision of 0.717, and an accuracy of 0.777. The RF model demonstrated an AUC of 0.839, an average precision of 0.747, and an accuracy of 0.789. The ANN model achieved an AUC of 0.835, an average precision of 0.733, and an accuracy of 0.780.

Including post-admission parameters significantly enhanced the models’ performance. In the test sets, the LR model’s AUC increased to 0.927, with an average precision of 0.881 and an accuracy of 0.875. The DT model achieved an AUC of 0.907, an average precision of 0.848, and an accuracy of 0.851. The RF model’s AUC rose to 0.926, with an average precision of 0.877 and an accuracy of 0.861. The ANN model again demonstrated a slightly higher performance, with an AUC of 0.941, an average precision of 0.898, and an accuracy of 0.884.

In addition to pre-admission features (*n* = 65), the inclusion of post-admission features (*n* = 37) improved the AUC for the ANN model from 0.835 to 0.941, RF from 0.839 to 0.926, DT from 0.825 to 0.907, and LR from 0.831 to 0.927. The average AUC improvement across all models was 0.089 ± 0.012, showing consistent enhancements across all models with post-admission data.

A stratified analysis of mortality rates was conducted to assess the potential impact of the COVID-19 pandemic relevant to the current literature. The 1-month mortality rate increased from 20.9% in the pre-COVID period to 24.57% in the post-COVID period (*p* < 0.001). Similarly, the 1-year mortality rate rose from 30.85% to 35.55% (*p* < 0.001). Due to the unavailability of patient-level COVID-19 data, pandemic-specific effects were not incorporated into the predictive models.

### 3.4. Significance Analysis

As demonstrated in Table 4, in both 1-month and 1-year mortality predictions, several key prognostic factors were identified as highly significant (*p* < 0.001). Among these, age emerged as one of the strongest predictors, with older patients showing a significantly increased risk of mortality at both time points. Cardiopulmonary arrest and endotracheal intubation at ED was another critical factor, demonstrating a strong association with mortality, further emphasizing the severity of the initial clinical presentation. The placement of an EVD within the first 72 h, while not a significant predictor for 1-month mortality, became a meaningful factor for 1-year mortality. Elevated glucose and lactate levels were also important markers of poor outcomes, while a higher white blood cell count (WBC) was strongly correlated with increased mortality risk. Pre-existing comorbidities, such as chronic heart disease, hypertension, and chronic kidney disease, were consistently significant across both 1-month and 1-year predictions, highlighting the influence of underlying health conditions on patient outcomes.

Additionally, postoperative complications, including sepsis, epilepsy, pneumonia, and deep vein thrombosis (DVT), were crucial determinants of mortality. Notably, postoperative pneumonia was among the most significant complications affecting survival. These findings underscore the importance of incorporating both pre- and post-admission data to enhance the predictive accuracy of mortality models in SAH patients, particularly in high-risk individuals with severe presentations and multiple comorbidities.

## 4. Discussion

Our study successfully developed and validated ML models to predict first-month and first-year mortality in patients with SAH using the comprehensive nationwide EHR system in Türkiye. Previous research has extensively explored various ML models for this purpose, and our findings align with and expand upon these efforts [22,23,24]. Unlike prior studies, our research utilizes Türkiye’s nationwide EHR system, marking the first extensive study of its kind within this unique national health information platform in SAH patients. Additionally, by focusing on first-month and first-year mortality and employing various advanced ML techniques, our study provides novel insights and contributes to the literature.

Our study utilized two distinct datasets and models trained on pre-admission and combined pre- and post-admission features. This approach serves two purposes: first, to enable early mortality prediction during hospital admission, facilitating timely discussions and planning with patients and their families; and second, to assess the impact of treatment decisions on mortality and morbidity, providing valuable insights into post-treatment outcomes. Including post-admission parameters substantially improved the performance of machine learning models in predicting mortality for SAH patients. For example, the ANN model’s AUC for 1-month mortality increased from 0.850 to 0.946 in the test set when incorporating post-admission data, with notable improvements in accuracy, precision, and recall. These enhancements underscore the importance of comprehensive data integration—from initial presentation through post-admission care—in improving prognosis predictions. Additionally, post-admission complications such as epilepsy, stroke, and pneumonia emerged as critical factors influencing survival. The high prevalence of epilepsy diagnoses may be attributed to preventive antiepileptic prescriptions, often entered as diagnoses. Early EVD placement, while not significant for 1-month mortality, became a key predictor for 1-year outcomes, reflecting its association with neurological deterioration and long-term survival. These findings highlight the value of a holistic clinical approach, particularly in neurosurgical settings, where detailed patient management strategies can meaningfully impact outcomes.

While our study primarily emphasizes the statistical methods and the overall utility of the models, the key predictors identified through our analysis deserve special attention. Factors such as age, cardiopulmonary arrest, and endotracheal intubation status emerged as significant predictors of both short- and long-term mortality. Older patients and those requiring intubation or experiencing cardiopulmonary arrest typically have less physiological reserve to recover from surgery and subsequent complications [25,26,27]. Furthermore, laboratory markers like elevated glucose and lactate levels, as well as high WBC counts, were strongly correlated with poor outcomes, reflecting the severity of the initial clinical presentation. Post-admission complications, including pneumonia, sepsis, and epilepsy, further underscored the importance of continuous monitoring and management in improving survival rates. Integrating comprehensive data from initial presentation through post-admission care allows for more precise risk stratification and targeted interventions, leading to better patient management and clinical outcomes. The improvement in model performance with post-admission data underscores the necessity of continuous monitoring and management in neurosurgical and intensive care settings. While objective clinical scales like the Fisher grade, WFNS, Hunt and Hess, and Glasgow Coma Scale provide valuable insights [28], our study demonstrates that comprehensive tabular data can effectively serve as a proxy for these objective measures. Despite the absence of these specific scales, our extensive data analysis achieved superior predictive performance, exceeding the standard range of AUCs reported in studies utilizing these clinical scales. This highlights the enhanced prognostic power of integrating extensive pre- and post-admission big data in SAH outcome predictions. Utilizing our ML algorithms, we achieved high predictive accuracy, highlighting our dataset’s potential to capture the essential prognostic elements of these scales indirectly. This approach allows for a reliable and robust prediction model, which is especially valuable in clinical settings where these specific scales may not be readily available or consistently applied.

Interestingly, our study demonstrated a significant rise in mortality rates, with 1-month mortality increasing from 20.9% pre-COVID to 24.57% post-COVID and 1-year mortality rising from 30.85% to 35.55% (*p* < 0.001). Consistent with our findings, an increase in SAH mortality during the COVID-19 pandemic has been reported; this could potentially be attributed to delayed presentation, worse neurological status, associated comorbidities, and decreased hospital resource utilization [29,30,31,32,33,34]. Due to the unavailability of patient-level COVID-19 data, pandemic-specific effects were not incorporated into our predictive models.

The performance comparison revealed that the ANN and RF models achieved the highest accuracy and AUC scores, likely due to their ability to capture complex relationships within the data. ANN’s deep learning architecture facilitates extracting intricate features, resulting in superior predictive accuracy. However, its clinical applicability may be limited by its computational complexity, the need for technical expertise, and substantial processing power. Conversely, LR offers high precision and AUC with simplicity and interpretability, making it suitable for resource-limited settings. DT demonstrated competitive performance. LR provides similar success rates to ANN while being less complex and more interpretable, making it a suitable option for practical implementation, as shown in Figure 1 and Figure 2. Additionally, as was in the case in our study, a significance analysis of the features can be conducted to further validate their importance in the model.

Recent research has demonstrated that ML algorithms can accurately predict in-hospital mortality and functional outcomes [19,35]. These models often outperform traditional statistical methods and can match clinicians’ predictions [36]. The multicenter retrospective cohort studies have effectively utilized ML to predict prognostic features and identify poor prognostic conditions related to SAH, such as DCI [15,37]. These studies have demonstrated the utility of various ML algorithms, including RF, gradient boosting DT, support vector machines, and DT, in predicting DCI and other complications and poor prognostic outcomes following SAH [7,10,11,38]. These studies highlighted the importance of incorporating clinical variables to enhance predictive accuracy, a methodology we also adopted and found effective. Recent studies have reported average AUC values between 0.80 and 0.87 for predicting outcomes in SAH, demonstrating the efficacy of ML in enhancing prognostic accuracy. In comparison, our models achieved superior performance, with the ANN model reaching an AUC of 0.945 for 1-month mortality and 0.937 for 1-year mortality. This improvement underscores the impact of incorporating pre- and post-admission features and big data analysis.

Key predictors include age, serum glucose, neutrophil-to-lymphocyte ratio, and established clinical scales such as WFNS and Fisher’s scale [39]. Some researchers have developed simplified scorecards based on ML models to facilitate bedside use [6]. Additionally, the others applied ML to a nationwide electronic health record (EHR) database to predict mortality and poor outcomes in SAH patients. This demonstrates that logistic regression and other ML models could achieve high predictive performance when leveraging comprehensive clinical datasets [19,40].

Our research is consistent with the findings of Farooqi et al., who emphasized the potential of AI and ML in refining grading and outcome predictions for aneurysmal SAH. They highlighted that integrating extensive datasets from nationwide EHR could significantly improve the accuracy and applicability of predictive models [41]. Similarly, other recent studies underscored the critical role of early clinical indicators and the application of ML algorithms in predictive modeling for SAH patients [42,43,44]. Integrating pre- and post-admission variables into our ML models significantly enhanced their predictive performance. This approach aligns with Shu et al.’s findings, demonstrating the crucial role of post-admission factors in accurately predicting patient outcomes after SAH using Shapley Additive Explanations (SHAPs) analysis to enhance model interpretability [45]. Our results underscore the importance of comprehensive data collection throughout the patient’s clinical journey, from initial presentation to post-admission care.

A major strength of our study is the utilization of both the e-Nabız and MERNİS systems. The e-Nabız system provides a comprehensive and representative dataset from all hospitals in Türkiye, providing robust and reliable clinical data. Similarly, the MERNİS system offers accurate and dependable mortality data. Together, these national platforms contribute significantly to the validity and generalizability of our findings. Applying advanced ML techniques, including ANN, DT, RF, and LR, also allowed for high predictive accuracy.

However, the study’s retrospective design and potential biases in data collection are notable limitations. Despite rigorous data preprocessing and imputation techniques, some residual confounding factors may exist. Moreover, excluding patients with incomplete records may have introduced selection bias. A further limitation of our study is the absence of an objective scale to assess patients’ conditions at presentation (such as Hunt-Hess, mRS, or GCS scores). To address this, we categorized patients into “good” and “poor” presentation groups based on their first presentation in ED, such as arrest history, intubation status, and later clinical and operational factors like complications, epilepsy, or sepsis. This proxy measure allowed us to account for the severity of the presentation indirectly, although it does not fully replace standardized clinical scoring systems. Future studies should focus on the prospective validation of the models and consider incorporating additional clinical, imaging, and laboratory variables to enhance predictive accuracy.

Future research should validate our predictive models prospectively in different clinical settings to confirm their generalizability and reliability. Integrating more granular clinical data and molecular and biochemical markers could provide deeper insights into SAH prognosis and improve model performance. Exploring other ML algorithms and ensemble methods may yield better predictive accuracy. Investigating the impact of incorporating continuous monitoring data, such as real-time physiological parameters, could enhance the timeliness and precision of outcome predictions [46]. Moreover, future nationwide studies should include robust scales such as WFNS, Hunt and Hess, and Fisher, which are unavailable in our cohort, to further refine the predictive models and improve their applicability.

## 5. Conclusions

Our study developed and validated ML models to predict 1-month and 1-year mortality in patients with SAH using data from Türkiye’s unique nationwide EHR system. Notably, including pre- and post-admission parameters significantly enhanced the models’ predictive performance, with ANN achieving the highest accuracy and AUC. However, LR also demonstrated strong performance, closely matching ANN while being more interpretable and more accessible to apply in clinical practice. Key predictors included age, cardiopulmonary arrest and/or endotracheal intubation, abnormal laboratory results (such as glucose and lactate levels) on presentation, and post-admission complications such as pneumonia and sepsis, which significantly impacted both short- and long-term mortality. These findings offer valuable clinical implications for the early identification of high-risk SAH patients, enabling timely and personalized interventions. Moreover, this study is the first comprehensive SAH analysis using a nationwide dataset in the Turkish population, underscoring its unique contribution to the field. Future research should focus on the prospective validation of these models and further integration of clinical variables to enhance predictive accuracy, potentially improving patient outcomes and optimizing resource allocation in neurosurgery.

## Figures and Tables

**Table 1:** Characteristics of patients: demographics, clinical presentation, interventions, and outcomes.

Variable	Value
Number of patients	29,274
Gender	
Female	14,002 (47.8)
Male	15,272 (52.2)
Age	56.23 ± 16.45
	57 [46–68]
Time from initial emergency visit to CT scan, minutes	12 [4–114]
Time from initial emergency visit to diagnosis, hours	3 [0–23]
Intubation in the first 24 h	10,017 (34.2)
EVD in the first 72 h	2840 (9.7)
Lab values in the initial emergency visit	
Hemoglobin (g/dL)	13 [11.4–14.4]
RBC (×10^3^ cells/μL)	4.5 [4–4.9]
WBC (×10^3^ cells/μL)	12.01 [9.2–15.6]
Platelets (×10^3^ cells/μL)	230 [184–282]
Lymphocyte (×10^3^ cells/μL)	4.3 [1.4–10.5]
Neutrophil (×10^3^ cells/μL)	22.34 [10–82.9]
Sodium (mmol/L)	138.8 [136–141]
Glucose (mg/dL)	131 [105–167]
Lactate (mmol/L)	1.91 [1.3–3.2]
Comorbidities	
Type 2 Diabetes Mellitus (DM)	7460 (25.5)
Hyperlipidemia	6968 (23.8)
Atherosclerosis	356 (1.2)
Hypertension	16,313 (55.7)
Acute ischemic heart disease	2994 (10.2)
Chronic ischemic heart disease	7845 (26.8)
Cerebrovascular disease	5565 (19)
Peripheral artery disease	2563 (8.8)
Renal failure	1402 (4.8)
Malignancy	1118 (3.8)
Inflammatory disease	1110 (3.8)
Rheumatic heart diseases	640 (2.2)
Liver disease	836 (2.9)
Chronic obstructive pulmonary disease (COPD)	3529 (12.1)
Other aneurysms	682 (2.3)
Obesity	553 (1.9)
Pregnancy	788 (2.7)
Neurocutaneous disorders	8 (0.02)
Coagulation disorders	554 (1.9)
Primary intervention for aneurysm	
Yes	11,068 (37.8)
Clipping	6643 (22.7)
Coiling	4425 (15.1)
No	18,206 (62.2)
Time from initial emergency visit to intervention for aneurysm, days	1.95 [0.75–4.75]
Clipping	1.95 [0.77–4.4]
Coiling	2.07 [0.8–5.8]
Facilities in which intervention for aneurysm was performed	
Government-owned hospitals	5461 (49.3)
University hospitals	3396 (30.7)
Private hospitals	1674 (15.1)
Private university hospitals	537 (4.6)
Interventions for SAH complications	
Yes	8323 (28.4)
Decompressive craniectomy	648 (2.2)
Epidural hematoma evacuation	125 (0.4)
Subdural hematoma evacuation	938 (3.2)
Intracerebral hemorrhage evacuation	1233 (4.2)
CSF drainage	
EVD and/or ELD placement	3090 (10.6)
VPS placement	345 (1.2)
Tracheostomy	747 (2.6)
PEG placement	522 (1.8)
No	20,951 (71.6)
Length of hospitalization, days	19.01 ± 15.94
	15.9 [8.6–24.8]
Emergency revisit within 90 days of discharge	5100 (17.4)
Death within 7 days	2757 (9.4)
Death within 30 days	6668 (22.8)
Death within the first year	9737 (33.3)
Post-treatment complications	
Acute respiratory distress syndrome (ARDS)	331 (1.1)
Respiratory failure	4502 (15.4)
Acute ischemic heart disease	1781 (6.1)
Sepsis	1380 (4.7)
Meningitis	294 (1)
Encephalitis	48 (0.2)
Intracranial and intraspinal abscess	69 (0.2)
Urinary tract infection (UTI)	3691 (12.6)
Epilepsy	8220 (28.1)
Hydrocephalus	960 (3.3)
Cerebral edema	1937 (6.6)
Pulmonary thromboembolism (PTE)	506 (1.7)
Deep vein thrombosis (DVT)	1137 (3.9)
Pneumonia	4914 (16.8)
Paralysis	3148 (10.8)
Status epilepticus	216 (0.7)
Decubitus ulcer	1382 (4.7)
Cerebral Ischemia	1191 (4.1)

**Table 2:** Machine learning prediction results for first-month mortality in SAH patients.

			Machine Learning Methods			
Input Features	Sample	Metric	Logistic Regression	Decision Tree	Random Forest	Artificial Neural Network
**Pre-admission**	Training	AUC	0.845	0.861	0.882	0.855
		Average Precision	0.654	0.692	0.750	0.673
		Accuracy	0.830	0.842	0.852	0.834
	Test	AUC	0.849	0.835	0.855	0.850
		Average Precision	0.651	0.636	0.667	0.649
		Accuracy	0.832	0.826	0.835	0.829
**Pre-admission** **+** **Post-admission**	Training	AUC	0.940	0.937	0.942	0.952
		Average Precision	0.825	0.797	0.840	0.858
		Accuracy	0.895	0.886	0.892	0.906
	Test	AUC	0.942	0.916	0.931	0.946
		Average Precision	0.835	0.755	0.813	0.844
		Accuracy	0.901	0.885	0.893	0.905

**Table 3:** Machine learning prediction results for first-year mortality in SAH patients.

			Machine Learning Methods			
Input Features	Sample	Metric	Logistic Regression	Decision Tree	Random Forest	Artificial Neural Network
**Preadmission**	Training	AUC	0.837	0.853	0.863	0.846
		Average Precision	0.744	0.772	0.796	0.755
		Accuracy	0.786	0.801	0.807	0.793
	Test	AUC	0.831	0.825	0.839	0.835
		Average Precision	0.733	0.717	0.747	0.733
		Accuracy	0.782	0.777	0.789	0.780
**Pre-admission** **+** **Post-admission**	Training	AUC	0.929	0.933	0.939	0.949
		Average Precision	0.885	0.889	0.898	0.914
		Accuracy	0.874	0.864	0.865	0.893
	Test	AUC	0.927	0.907	0.926	0.941
		Average Precision	0.881	0.848	0.877	0.898
		Accuracy	0.875	0.851	0.861	0.884

**Table 4:** Results of significance analysis for key predictors.

	First Month Prediction				First Year Prediction			
	Estimate	Std.	Error	Pr (>|z|)	Estimate	Std.	Error	Pr (>|z|)
Age	2.350475	0.151	15.48	<0.001	4.188651	0.143	29.097	<0.001
Cardiopulmonary Arrest	4.138183	0.234	17.613	<0.001	4.488835	0.328	13.684	<0.001
Endotracheal Intubation	−2.117409	0.234	−9.026	<0.001	−2.797108	0.328	−8.524	<0.001
EVD (First 72 h)	−0.10164	0.116	−0.87		−0.452086	0.107	−4.187	<0.001
Glucose	5.339468	0.599	8.913	<0.001	6.429198	0.582	11.036	<0.001
Lactate	5.014475	1.330	3.77	<0.001	4.807589	1.233	3.896	<0.001
Neutrophil	1.006599	0.325	3.093	<0.01	0.228911	0.305	0.748	
Basophil	3.633494	1.309	2.774	<0.01	2.950229	1.216	2.425	<0.05
White Blood Cell Count	7.284652	0.523	13.917	<0.001	6.646928	0.510	13.023	<0.001
Eosinophil	−3.618516	1.089	−3.321	<0.001	−2.604965	0.879	−2.961	<0.01
Hematocrit	−0.351574	0.129	−2.719	<0.01	−0.339932	0.117	−2.901	<0.01
Erythrocyte	0.246341	0.481	0.511		−1.133348	0.436	−2.598	<0.01
Mean Platelet Volume	0.528842	0.239	2.209	<0.05	0.635336	0.221	2.872	<0.01
Platelet Distribution Width	1.124736	0.248	4.528	<0.001	0.835278	0.225	3.710	<0.001
Platelet	−0.409396	0.168	−2.424	<0.05	−0.413981	0.153	−2.705	<0.01
Platelet to WBC Ratio	−5.862795	0.908	−6.452	<0.001	−1.629248	0.736	−2.213	<0.05
Pre-existing Hypertension	0.290937	0.053	5.415	<0.001	0.31136	0.047	6.528	<0.001
Pre-existing Chronic Heart Disease	0.227944	0.065	3.496	<0.001	0.408031	0.058	6.960	<0.001
Pre-existing Stroke	0.089569	0.063	1.409		0.327823	0.056	5.841	<0.001
Pre-existing Pulmonary Arterial Hypertension	0.188741	0.081	2.313	<0.05	0.20692	0.072	2.844	<0.01
Pre-existing Chronic Kidney Disease	0.583693	0.10	5.832	<0.001	0.627275	0.092	6.812	<0.001
Pre-existing Malignancy	0.124178	0.110	1.123		0.831856	0.095	8.754	<0.001
Pre-existing Rheumatic Vascular Disease	0.574583	0.146	3.935	<0.001	0.212816	0.133	1.596	
Pre-existing Chronic Liver Failure	0.284246	0.088	3.212	<0.01	0.558313	0.081	6.883	<0.001
Pre-existing Epilepsy	0.272037	0.118	2.301	<0.05	0.736211	0.098	7.444	<0.001
Pre-existing Hemiplegia/Paraplegia	0.769522	0.170	4.51	<0.001	0.829921	0.146	5.677	<0.001
Institution Type	−3.231709	0.433	−7.462	<0.001	−3.539716	0.630	−5.618	<0.001
Procedure-related Complications	−0.005564	0.143	−0.039		1.231947	0.114	10.728	<0.001
Decompressive Craniectomy	0.941764	0.174	5.404	<0.001	0.446239	0.157	2.839	<0.01
Subdural Hematoma	−0.097749	0.180	−0.54		−0.704535	0.149	−4.727	<0.001
Intracerebral Hemorrhage	0.598519	0.159	3.741	<0.001	0.064577	0.137	0.469	
Tracheostomy	−1.026597	0.137	−7.454	<0.001	0.510161	0.114	4.447	<0.001
Percutaneous Endoscopic Gastrostomy	−2.665337	0.257	−10.352	<0.001	0.283103	0.129	2.190	<0.05
EVD (anytime during the hospitalization)	0.726111	0.181	3.99	<0.001	0.470255	0.161	2.916	<0.01
Postoperative Sepsis	−0.099639	0.106	−0.932		1.04247	0.088	11.825	<0.001
Postoperative Epilepsy	−2.163765	0.081	−26.651	<0.001	−1.755134	0.054	−32.30	<0.001
Postoperative Hemiplegia/Paraplegia	−1.732357	0.141	−12.216	<0.001	−1.111446	0.077	−14.29	<0.001
Postoperative Hydrocephalus	−0.821742	0.169	−4.856	<0.001	−0.388568	0.115	−3.351	<0.001
Postoperative Brain Edema	0.345695	0.080	4.286	<0.001	0.422068	0.075	5.598	<0.001
Postoperative Pulmonary Thromboembolism	−0.833607	0.264	−3.155	<0.01	−0.040277	0.157	−0.256	
Postoperative Deep Vein Thrombosis	−1.830364	0.277	−6.607	<0.001	−0.836692	0.128	−6.490	<0.001
Postoperative Stroke	−0.662205	0.044	−15.043	<0.001	−0.572621	0.040	−14.004	<0.001
Postoperative Pneumonia	−1.23275	0.051	−24.065	<0.001	−0.979274	0.042	−22.84	<0.001
Postoperative Urinary Tract Infection (UTI)	−1.681855	0.101	−16.526	<0.001	−1.311541	0.062	−20.96	<0.001
Postoperative Decubitus Ulcers	−2.255048	0.221	−10.172	<0.001	0.421185	0.092	4.552	<0.001
Postoperative Respiratory Failure	0.479471	0.059	8.024	<0.001	0.930121	0.053	17.294	<0.001
Postoperative Chronic Heart Disease	−1.715899	0.081	−20.984	<0.001	−1.838105	0.062	−29.58	<0.001
30-day Emergency Re-admission After Discharge	−1.259397	0.123	−10.163	<0.001	0.31285	0.106	2.925	<0.01

## Data Availability

The data supporting the findings of this study, including all images and datasets, are available from the corresponding author, Sahin Hanalioglu, upon reasonable request. No additional external data repositories were used, and the data are not publicly archived due to the nature of this study. All relevant data are contained within the article.
